# Redox active dendronized polystyrenes equipped with peripheral triarylamines

**DOI:** 10.3762/bjoc.10.326

**Published:** 2014-12-22

**Authors:** Toshiki Nokami, Naoki Musya, Tatsuya Morofuji, Keiji Takeda, Masahiro Takumi, Akihiro Shimizu, Jun-ichi Yoshida

**Affiliations:** 1 Department of Synthetic Chemistry and Biological Chemistry, Graduate School of Engineering, Kyoto University, Nishikyo-ku, Kyoto 615-8510, Japan

**Keywords:** carbocation, cross-coupling, dendrimer, dendronized polymer, redox

## Abstract

Dendronized polystyrene having peripheral bromo groups was prepared from the dendronization of unfunctionalized polystyrene with dendritic diarylcarbenium ions bearing peripheral bromo groups using the “cation pool” method. The palladium-catalyzed amination of the peripheral bromo groups with diarylamine gave dendronized polystyrene equipped with peripheral triarylamines, which exhibited two sets of reversible redox peaks in the cyclic voltammetry curves.

## Introduction

Assembling small functional molecules using dendrimers [1] and dendronized polymers [2–8] as scaffolds serves as a useful method for synthesizing organic functional materials having nanosize three-dimensional structures. Although there are many examples of redox-active dendrimers, including those equipped with ferrocene [9–10], triarylamines [11–14], and tetrathiafulvalene (TTF) derivatives [15–19], the corresponding dendronized polymers are rare [20]. One of the major reasons for this seems to be the difficulty in making such structures. However, redox-active dendronized polymers should provide more opportunities to form functional organic materials, and therefore, the development of efficient methods for the synthesis of redox-active dendronized polymers is highly desirable. Recently, we have developed a new method [21–26] for the synthesis of dendronized polymers [27] from the dendronization of unfunctionalized polystyrenes with electrogenerated dendritic diarylcarbenium ions. The simplicity and step economy of this method prompted us to synthesize dendronized polymers equipped with peripheral functional groups by the use of this method.

**Figure 1:**
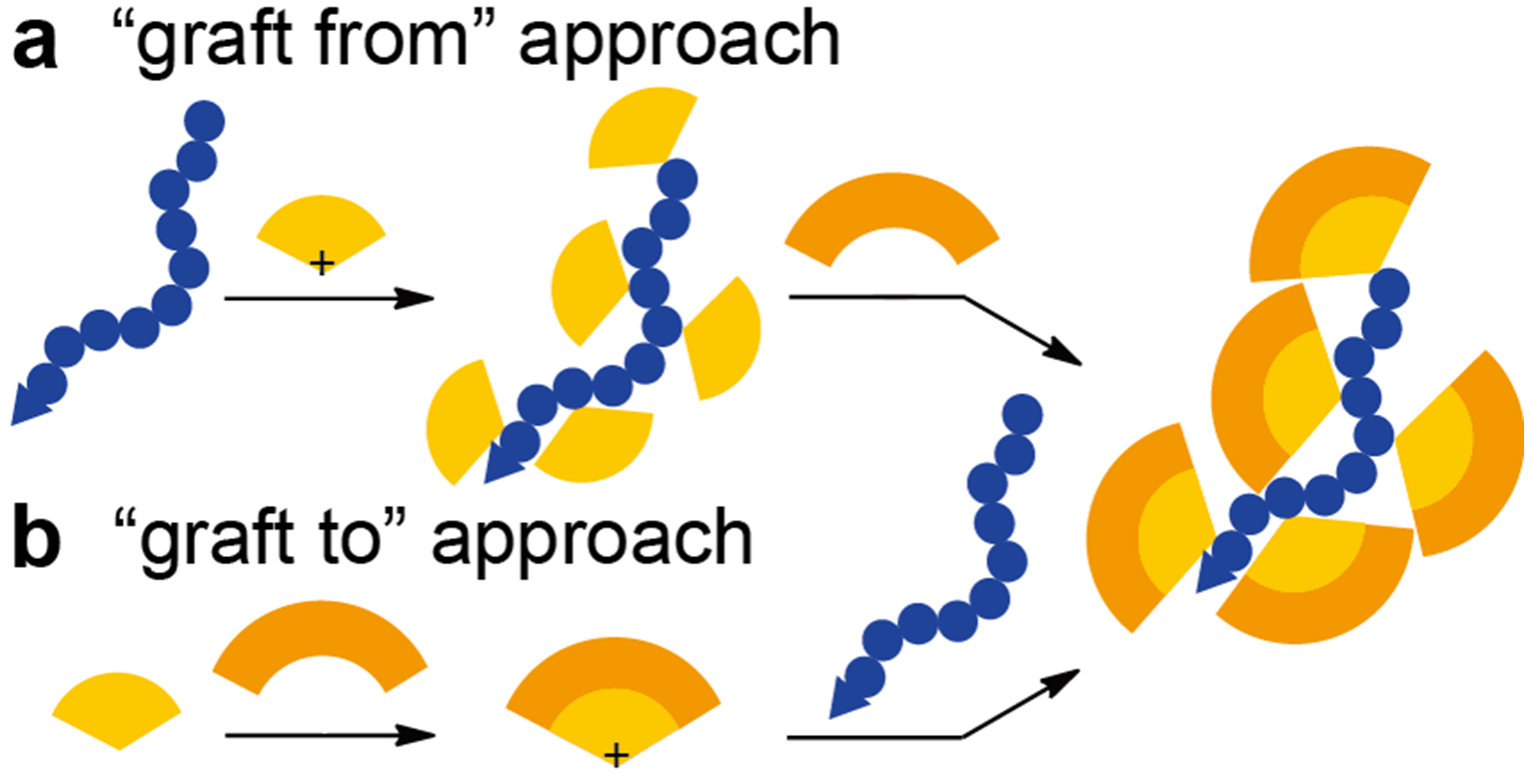
Two synthetic approaches toward the peripherally functionalized dendronized polystyrenes (blue dotted line = polystyrene, orange sector = dendron of the 1st generation, dark orange sector = dendron of the 2nd generation). (a) Peripheral functionalization of the dendronized polystyrene (“graft from” approach). (b) Dendronization with dendritic diarylcarbenium ions equipped with functional groups (“graft to” approach).

In principle, there are two synthetic approaches for synthesizing peripherally functionalized dendronized polystyrenes: (a) the functionalization of dendronized polystyrene (the “graft from” approach, Figure 1a); and (b) dendronization of polystyrenes with the dendritic carbocation equipped with functional groups (the “graft to” approach, Figure 1b). Both approaches have advantages and disadvantages. In the former case, more dendritic scaffolds can be introduced by functionalization, but structural inhomogeneity can occur from incomplete peripheral functionalization. In the latter approach, direct dendronization by the functionalized diarylcarbenium ions may be difficult, although the dendritic substituents would have a uniform structure. In this paper we report on the synthesis of redox-active dendronized polystyrenes via the peripheral modification of dendronized polystyrene [28].

## Results and Discussion

**Figure 2:**
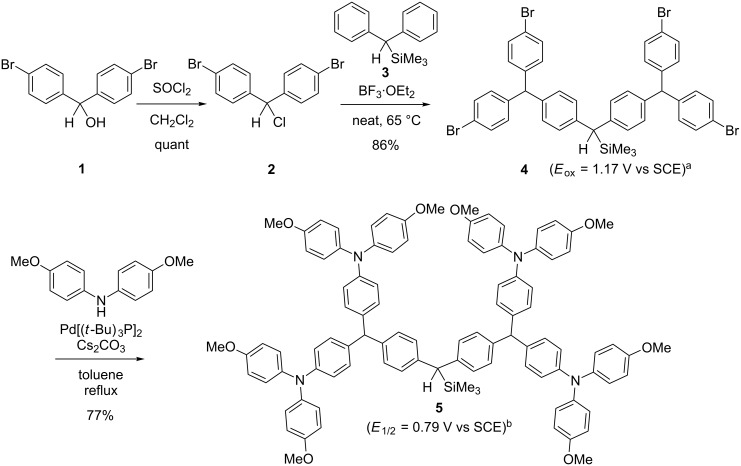
Preparation of the dendrimer having peripheral bromo groups and their conversion to diarylamino groups. ^a^Oxdation potential (*E*_ox_). ^b^Redox potential (*E*_1/2_).

Dendrimer **4**, having a bromo functionality was prepared on a multigram scale, as shown in Figure 2. Di(*p*-bromophenyl)carbinol (**1**) was treated with SOCl_2_ to obtain di(*p*-bromophenyl)methyl chloride (**2**) in quantitative yield. A Friedel–Crafts-type alkylation [29] of diphenylsilane **3** with **2** in the presence of boron trifluoride etherate as a Lewis acid gave **4** in 86% yield. The oxidation potential of **4** (*E*_ox_ = 1.17 V vs SCE) is slightly lower than that of the fluorine analogue (*E*_ox_ = 1.25 V vs SCE) which was used as a precursor of the dendritic cation in our previous work [25], indicating that bromine analogue **4** can act as a precursor of the dendritic cation.

The functionalization of **4** was examined before studying the functionalization of dendronized polystyrene. Thus, di(*p*-methoxyphenyl)amino groups were introduced to **4** using a Buchwald–Hartwig amination [11]. The choice of a base is crucial for this transformation, and the transformation was successfully carried out using Cs_2_CO_3_ as a base and Pd[P(*t*-Bu)_3_]_2_ as a catalyst [30] to obtain the dendrimer **5** having peripheral diarylamino groups in a yield of 77%. Compound **5** can serve as the precursor of dendritic diarylcarbenium ions having peripheral triarylamine structures. However, its redox potential (*E*_1/2_ = 0.79 V vs SCE) is much lower than the oxidation potential of dendrimer **4**, indicating that the triarylamine moiety is oxidized before the benzylsilane moiety. Using the “graft to” approach to synthesize peripherally functionalized dendronized polystyrene (Figure 1b) employing **5** as a precursor of the dendritic carbocation was unsuccessful. Low-temperature electrochemical oxidation of dendrimer **5** using the “cation pool” method [31–47] did not give the corresponding dendritic diarylcarbenium ion even, when subjected to excess capacitance (up to 5.0 *F*/mol).

**Figure 3:**
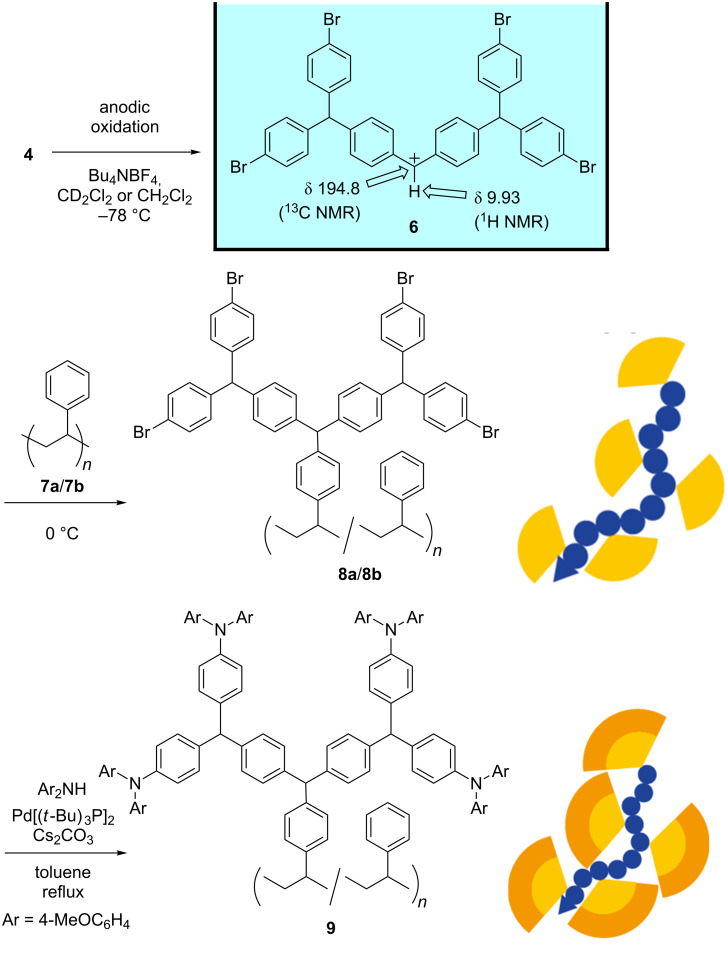
Preparation of dendronized polystyrenes having peripheral diarylamino groups.

Next, we examined the “graft-from” approach (Figure 3). The low-temperature electrochemical oxidation of dendrimer **4** using the “cation pool” method was performed in CD_2_Cl_2_, and the resulting anodic solution was transferred to NMR tubes. Low-temperature NMR analysis indicated that an accumulation of dendritic diarylcarbenium ion **6** in the solution had occurred. The chemical shift of the cationic carbon (^13^C NMR δ 194.8) and that of the proton attached to the cationic carbon (^1^H NMR δ 9.93) indicated that there was no interaction between the cationic carbon and peripheral bromo groups, because the chemical shifts were almost identical to those of the fluorine analogue (^13^C NMR δ 194.4, ^1^H NMR δ 9.92) [25].

**Figure 4:**
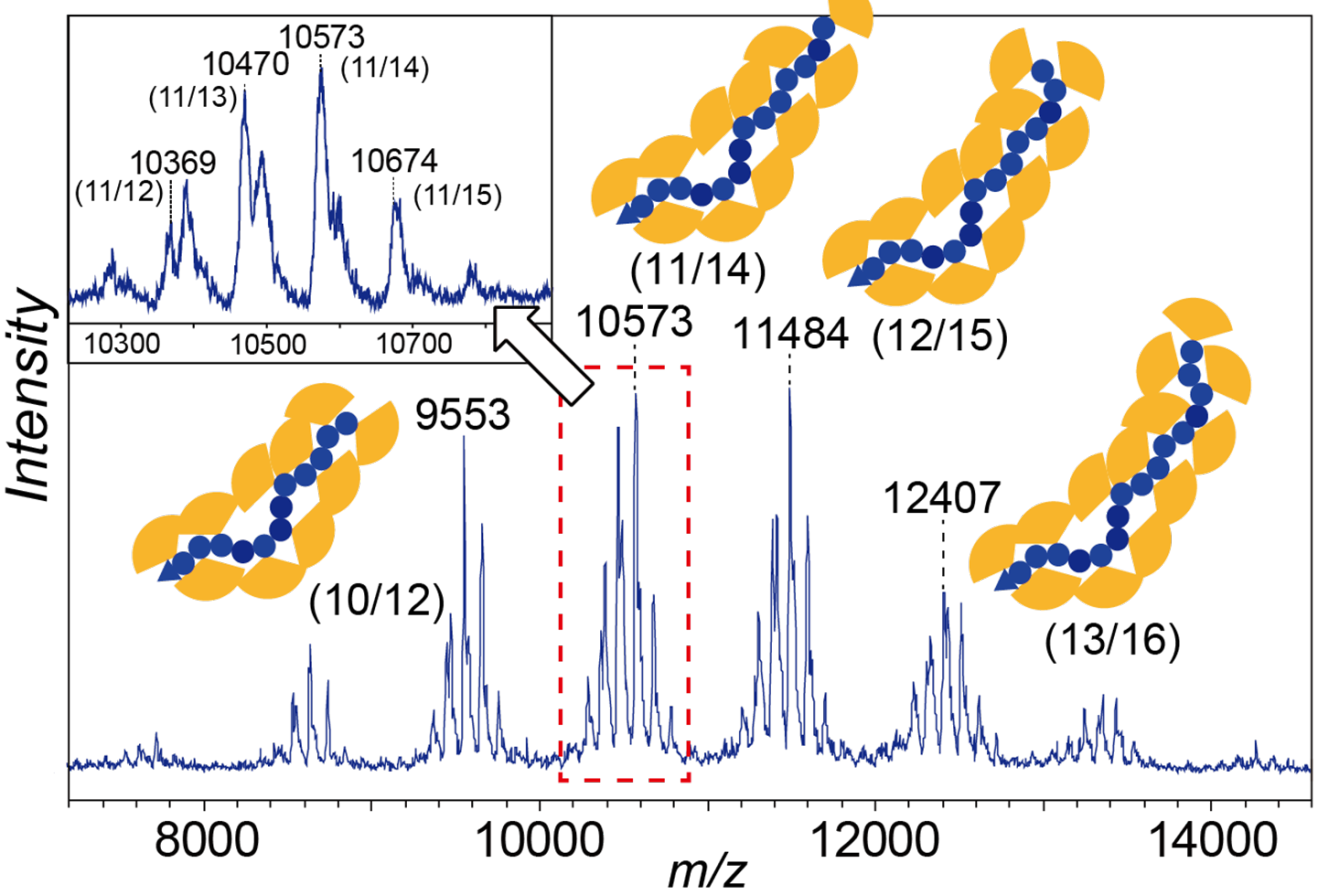
MALDI–TOF MS analysis of the dendronized polystyrene with peripheral bromo groups.

The reaction of **6** (generated on a preparative scale in CH_2_Cl_2_) with a low-molecular weight polystyrene **7a** (*M*_n_ = 1,580, polydispersity index (PDI) = 1.04, 22 mg) was performed at 0 °C (Figure 3). The resulting dendronized polystyrene **8a** was characterized using MALDI–TOF MS analysis. Six peak groups were observed, as shown in Figure 4. The peak occurring at 10,573 Da [M + Ag^+^] is derived from 11 dendritic substituents (815 Da × 11), 14 styrene units (103 Da × 11 + 104 Da × 3), and a butyl group (58 Da), which was derived from the initiator at the end of the polystyrene. The broader peaks seem to be attributable to two isotopes of the bromo groups (79 and 81 Da), and the small peak separation 101–103 Da is consistent with the molecular weight of styrene monomer (104 Da). The MS analysis indicated that **6** reacted with about 80% of the phenyl groups on polystyrene **7a**. This ratio is consistent with the implemented rate (77%) calculated from the increase in weight of the polystyrene (22 mg to 150 mg).

In a similar manner, dendronized polystyrene **8b** was synthesized from polystyrene having a longer chain length **7b** (*M*_n_ = 9,300, PDI = 1.02). Using GPC analysis to estimate the molecular weight of the dendronized polymer was not appropriate, because both the *M*_n_ and *M*_w_ values of the obtained dendronized polystyrene **8b** were only twice that of the starting polystyrene **7b** (Table 1). SEC–MALLS analysis indicated that **6** reacted with about 70% of the phenyl groups on polystyrene **7b**. This ratio is slightly larger than the implemented rate (55%) calculated from the increase in weight of the polystyrene (156 mg to 820 mg).

The peripheral bromo groups of **8b** were converted to diarylamino groups using a Hartwig–Buchwald amination employing Pd[P(*t*-Bu)_3_]_2_ (10 mol %) and Cs_2_CO_3_ (12 equiv) to obtain **9**. Dendronized polystyrene **9** was also analyzed using GPC and SEC–MALLS measurements employing DMF as an eluent. The results are summarized in Table 1. GPC analysis indicated either a slight increase or no increase in molecular weight from the peripheral functionalization, but SEC–MALLS measurements showed an identifiable increase in the molecular weight (*M*_w_ = 62,000 to 226,000), which is more than double the theoretical value (*M*_w_ = 100,000 after 100% conversion of the peripheral bromo groups to diarylamino groups). Although the PDI did not change appreciably after dendronization (**7b** to **8b**), the PDI increased slightly after peripheral functionalization (**8b** to **9**). Presently, the ratio of methine protons in the focal points of the dendritic structure (δ 5.38–5.00, broad singlet, 3H) and the methyl protons of the peripheral methoxy groups (δ 3.80–3.40, broad singlet, 24H) in the ^1^H NMR spectra is the only evidence for the conversion of the peripheral bromo groups to diarylamino groups. The observed ratio was 1:8.4 (methane/methyl), which indicates a quantitative conversion (theoretical ratio is 1:8).

**Table 1:** Molecular weight analyses of the dendronized polystyrenes.

polymer	GPC			SEC-MALLS		

	*M*_n_	*M*_w_	PDI	*M*_n_	*M*_w_	PDI

**7b**^a^	9,300	9,490	1.02	–	–	–
**8b**	18,800	19,900	1.06	59,400	62,000	1.04
**9**	18,200	22,100	1.21	175,800	226,000	1.29

^a^Polymer **7b** was analyzed only by GPC.

**Figure 5:**
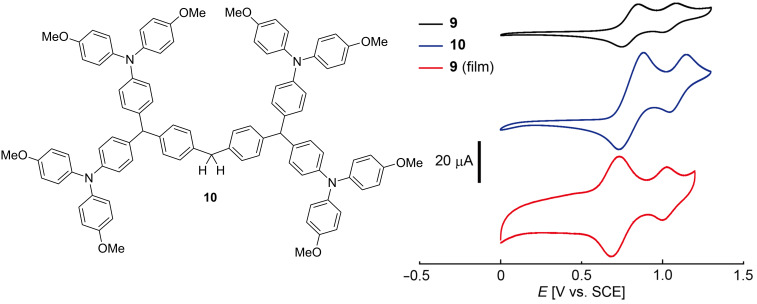
Cyclic voltammograms of dendronized polystyrene **9** (black line), model compound **10** (blue line), and the film of dendronized polystyrene **9** (red line). See the Experimental section for the details.

The redox behavior of **9** was studied by means of cyclic voltammetry (CV, Figure 5). Compound **9** showed two sets of reversible redox peaks (*E*_1/2_ = 0.80 and 1.04 V vs SCE), although it has been reported that di(*p*-methoxyphenyl)phenylamine shows a single redox peak in its CV curves [48]. Therefore, the two sets of redox peaks of **9** seem to be ascribable to the interaction of the initially formed radical cation from the triarylamine moiety with a neighboring neutral triarylamine moiety, which disfavors the second electron transfer from the latter, although the details of this reaction are not clear as yet [49–50]. This is consistent with the observation that dendrimer **10** prepared by desilylation of **5** followed by amination (see Supporting Information File 1 for preparative details) showed two reversible waves occurring at similar potentials (*E*_1/2_ = 0.81 and 1.10 V vs SCE) (Figure 5, blue line).

A film of dendronized polystyrene **9** was prepared on a Pt plate electrode as a film by drop casting of a CH_2_Cl_2_ solution of **9**. The redox behavior of the film of **9** on a Pt electrode was analyzed using CV (Figure 5, red line). Reversible CV cycles (*E*_1/2_ = 0.76 and 1.01 V vs SCE) were obtained in a mixed solvent (CH_3_CN/toluene 1:3) using Bu_4_NB(C_6_F_5_)_4_ as the electrolyte. The small shifts in the redox peaks from those obtained from solution may be attributable to the changes in solvent and electrolyte. The peak separations for a film of **9** (Δ*E* (*E*_ox_ – *E*_red_) = 52 and 27 mV) are significantly smaller than those observed for a solution of **9** (Δ*E* = 103 and 87 mV), indicating immobilization of **9** occurred on the surface of the electrode.

## Conclusion

In conclusion, redox-active dendronized polystyrene was successfully synthesized by peripheral functionalization of dendronized polystyrene having peripheral bromo groups with diarylamines. Thus, dendronized polystyrene having peripheral bromo groups may serve as a versatile precursor of dendronized polystyrenes equipped with various functional groups. Dendronized polystyrene having peripheral diarylamino groups showed reversible redox behavior in both solution and as a film deposited on an electrode. Further optimization of the present method and applications of peripheral functionalization of dendronized polystyrenes are under investigation in our laboratory.

## Experimental

### Electrochemical analyses

Electrochemical analysis was performed using an ALS/Chi700DS electrochemical analyzer. A saturated calomel electrode (SCE) (RE-2B, ALS Co. Ltd.) was used as the reference electrode.

The oxidation potential (*E*_ox_) of dendrimer **4** was measured using linear sweep voltammetry employing a gassy carbon (GC) rotating disk electrode (diameter = 3.0 mm, ALS Co. Ltd.) and a Pt wire counter electrode at room temperature. CH_2_Cl_2_ was used as a solvent and Bu_4_NBF_4_ (0.1 M) was used as a supporting electrolyte. The scan rate was 100 mV/s.

The redox potentials (*E*_1/2_) of **5** and **10**, and that of dendronized polystyrene **9** were measured by cyclic voltammetry (CV) using a GC working electrode (diameter = 3.0 mm, ALS Co. Ltd.) and a Pt wire counter electrode at room temperature. CH_2_Cl_2_ was used as a solvent and Bu_4_NBF_4_ (0.1 M) was used as a supporting electrolyte. The scan rate was 100 mV/s. The GC electrode was polished with alumina powder (0.05 μm) in water using a polishing pad, water-washed, and air-dried before use.

Cyclic voltammetry of a film of **9** deposited on a Pt plate electrode was measured as follows: Dendronized polystyrene **9** was deposited on a Pt plate electrode (1 × 1 cm^2^) as a film by drop casting of a CH_2_Cl_2_ solution of **9**, and the solvent was evaporated under reduced pressure to obtain the film of **9** attached to the Pt plate. The CV measurement was performed using the Pt plate with the film of **9** as the working electrode and a Pt wire counter electrode at room temperature. A mixer of CH_3_CN/toluene (1:3) was used a solvent and Bu_4_NB(C_6_F_5_)_4_ (0.1 M) as a supporting electrolyte. The scan rate was 100 mV/s.

## Supporting Information

File 1 Experimental procedures for the synthesis of new compounds and spectral data of new compounds including ^1^H NMR, ^13^C NMR, and HMQC spectra.
