# Supplementary material for: Redox active dendronized polystyrenes equipped with peripheral triarylamines
Source: Beilstein J Org Chem. 2014 Dec 22;10:3097–103. doi: 10.3762/bjoc.10.326 (PMC4311719; doi:10.3762/bjoc.10.326)

Supporting Information

for

Redox active dendronized polystyrenes equipped with peripheral triarylamines

Toshiki Nokami[§], Naoki Musya, Tatsuya Morofuji, Keiji Takeda, Masahiro Takumi, Akihiro Shimizu, and Jun-ichi Yoshida*

Address: Department of Synthetic Chemistry and Biological Chemistry, Graduate School of Engineering, Kyoto University, Nishikyo-ku, Kyoto 615-8510, Japan
Email: Jun-ichi Yoshida - yoshida@sbchem.kyoto-u.ac.jp

*Corresponding author

[§]Present address: Department of Chemistry and Biotechnology, Graduate School of Engineering, Tottori University, 4-101 Koyamachominami, Tottori 680-8552, Japan

Experimental procedures for the synthesis of new compounds and spectral data of new compounds including ^{1}H NMR, ^{13}C NMR, and HMQC spectra

Contents

1. General S2
2. Synthesis of the 1st generation dendrimer with peripheral bromo groups **4** S2
3. Hartwig-Buchwald amination to prepare the 2nd generation dendrimer **5** S2
4. NMR Analysis of dendritic diarylcarbenium ion **6** S3
5. Typical procedure for the synthesis of dendronized polystyrenes with peripheral bromo groups **8** S3–S4
6. Synthesis of the dendronized polystyrene equipped with peripheral triarylamines **9** S4
7. Synthesis of the 2nd generation dendrimer without trimethylsilyl group **10** S4–S5
8. References S5
9. ^{1}H NMR and ^{13}C NMR spectra S6–S14

1. General

^{1}H and ^{13}C NMR spectra were recorded on JEOL ECA-600P (^{1}H 600 MHz, ^{13}C 150 MHz). Chemical shifts are reported using the methine signal of $CHCl_3$ at δ 7.26 (^{1}H NMR) and δ 77.0 (^{13}C NMR) as internal standards. MALDI-TOF mass spectra were recorded on Bruker ultraflex III spectrometer. Analytical and Preparative gel permeation chromatography were performed on Shodex GPC-101 (an eluent: THF) and Japan Analytical Industry LC-918 (an eluent: $CHCl_3$) respectively. SEC-MALLS measurements were performed on SEC (Jasco PU-980 precision pump and RI-930 refractive index detector) coupled with a Dwan E instrument (Wyatt Technology; Ga-As laser, λ = 690 nm, an eluent: DMF containing 10 mM LiBr). Unless otherwise noted, all materials were obtained from commercial suppliers and used without further purification. Dichloromethane was washed with water, distilled from P_2O_5, redistilled from dried K_2CO_3 to remove a trace amount of acid, and stored over molecular sieves 4A. Supporting electrolyte $Bu_4NB(C_6F_5)_4$ was prepared from commercially available $NaB(C_6F_5)_4$ according to the reported procedure [1].

2. Synthesis of dendrimer with peripheral bromo groups 4

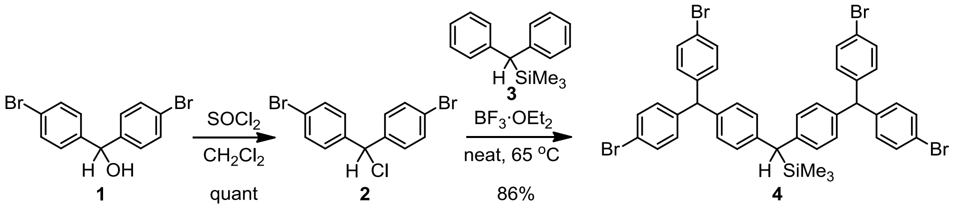

Compound **2** was prepared according to the procedure reported in the literature [2]. To a solution of 4,4-dibromobenzhydrol **1** (0.976 g, 2.85 mmol) in dichloromethane (7 mL) was added thionyl chloride (2.0 mL, ca. 28 mmol) dropwise at 0 °C, and the mixture was stirred at room temperature for overnight. The solvent was removed under reduced pressure, and the residue was extracted with ethyl acetate. The organic layer was washed with brine and dried on anhydrous $MgSO_4$. The solvent was removed under reduced pressure to afford the desired product **2** (0.989 g, quant), which was used for the next step without further purification.

Compound **2** (0.90 g, 2.5 mmol), trimethylsilyldiphenylmethane **3** [3] (0.22 g, 0.91 mmol) were dissolved in $BF_3 \cdot OEt_2$ (2.19 g, 15.4 mmol). After stirring for 24 h at 65 ^{o}C, the reaction was treated with iced water and extracted with AcOEt and dried over $MgSO_4$. After evaporation of the solvent, the crude product was purified with flash chromatography followed by reprecipitation from hexane to obtain **4** (689 mg, 86%). ^{1}H NMR (400 MHz, $CDCl_3$) δ 7.39 (d, J = 8.4 Hz, 8 H), 7.14 (d, *J* = 8.0 Hz, 4 H), 6.91-6.95 (m, 12 H), 5.38 (s, 2 H), 3.45 (s, 1 H), -0.01 (s, 9 H). ^{13}C NMR (150 MHz, $CDCl_3$) δ 142.57, 147.56, 141.2, 139.3, 131.5, 131.0, 129.1, 128.7, 120.4, 55.2, 45.3, -1.8. HRMS (FAB) *m/z* calcd for $C_{42}H_{36}Br_4SiAg^+$: 990.8371, found 990.8340.

3. Hartwig–Buchwald amination to prepare the 2nd generation dendrimer 5

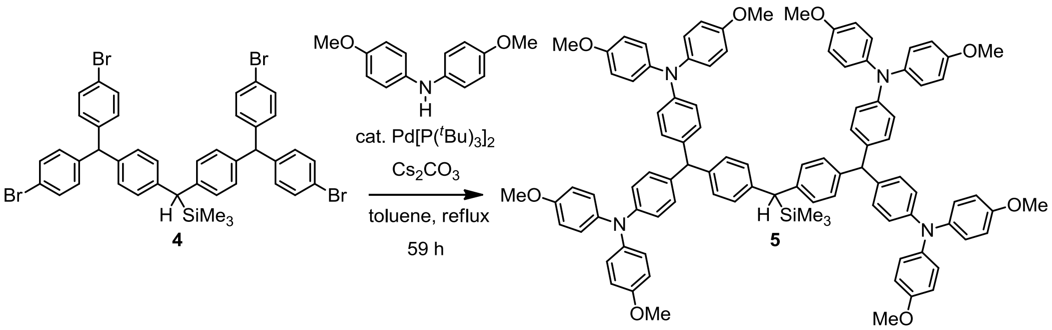

In a Schlenk flask, a mixture of $Pd[P(^tBu)_3]_2$ (0.06 mmol, 30 mg), Cs_2CO_3 (7.2 mmol, 2.34 g), bis(4-methoxyphenyl)amine (4.8 mmol, 1.10 g), and **4** (0.8 mmol, 716 mg) in toluene (10 mL) was stirred reflux for 59 h producing a heterogeneous mixture. The reaction mixture was treated with water and extracted with $CHCl_3$ and dried over $MgSO_4$. After evaporation of the solvent, the crude product was purified with GPC to obtain **5** (913 mg, 77%).^{1}H NMR (400 MHz, $CDCl_3$) δ 7.13 (d, *J* = 7.2 Hz, 4 H), 7.00-7.04 (m, 20 H), 6.89 (d, *J* = 8.4 Hz, 8 H), 6.83 (d, *J* = 8.8 Hz, 8 H), 6.80 (d, *J* = 8.4 Hz, 16 H), 5.29 (s, 2 H), 3.77 (s, 24 H), 3.44 (s, 1 H), -0.01 (s, 9 H).

^{13}C NMR ($CDCl_3$, 150 MHz) δ 155.5, 146.6, 141.2, 141.0, 140.5, 136.7, 129.8, 129.1, 128.3, 126.4, 126.3, 120.51, 120.47, 114.6, 114.5, 55.4, 55.0, 45.2, -1.7. HRMS (MALDI) *m/z* calcd for $C_{98}H_{93}N_4Si$ $[M+H]^+$: 1353.7086. found: 1353.7066.

4. NMR Analysis of dendritic diarylcarbenium ion 6

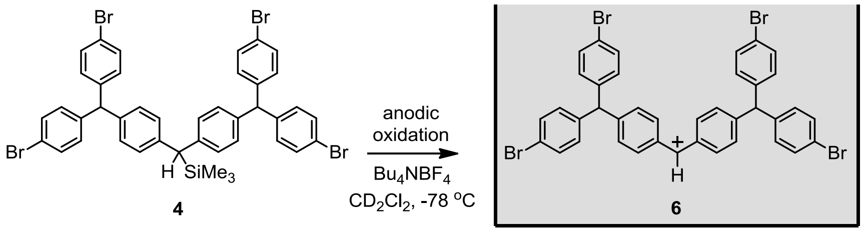

Dendritic cation **6** was prepared according to our previously reported procedure [4]. The anodic oxidation of **4** was carried out in an H-type divided cell (4G glass filter) equipped with a carbon felt anode (Nippon Carbon GF-20-P21E, ca. 160 mg, dried at 250 °C/1 mmHg for 2.5 h before use) and a platinum plate cathode (10 mm x 10 mm). In the anodic chamber were placed **4** (178 mg, 0.5 mmol) and 0.3 M Bu_4NBF_4/CD_2Cl_2 (5.0 mL). In the cathodic chamber were placed trifluoromethanesulfonic acid (70 μL, 0.80 mmol) and 0.3 M Bu_4NBF_4/CD_2Cl_2 (5.0 mL). The constant current electrolysis (5.0 mA) was carried out at -78 °C with magnetic stirring. After 2.5 F/mol of electricity was consumed, the reaction mixture of the anodic chamber was transferred to a 5 mm ϕ NMR tube with a septum cap under Ar atmosphere at -78 °C. The NMR measurement was carried out at -80 °C. Chemical shifts are reported using the methylene signal of CH_2Cl_2 at δ 5.32 (^1H NMR) and δ 53.8 (^{13}C NMR) as internal standards. Selected peaks for dendritic diarylcarbenium ion **6**; ^1H NMR (600 MHz, CD_2Cl_2) δ 9.93 (s, 1 H), 8.40 (d, *J* = 8.2 Hz, 2 H), 8.36 (d, *J* = 8.3 Hz, 2 H), 7.63 (d, *J* = 8.2 Hz, 2 H), 7.50 (d, *J* = 8.2 Hz, 2 H), 7.45 – 6.93 (m, 16 H), 5.76 (s, 2 H). ^{13}C NMR (150 MHz, CD_2Cl_2) δ 194.8 (cationic carbon), 165.7, 147.5, 140.3, 138.8, 135.3, 133.2, 132.3, 131.6, 130.9, 121.1, 55.9. The NMR data indicate that the rotation around the cationic carbon-aromatic carbon bond is restricted at -80 °C.

5. Typical procedure for the synthesis of dendronized polystyrenes with peripheral bromo groups 8

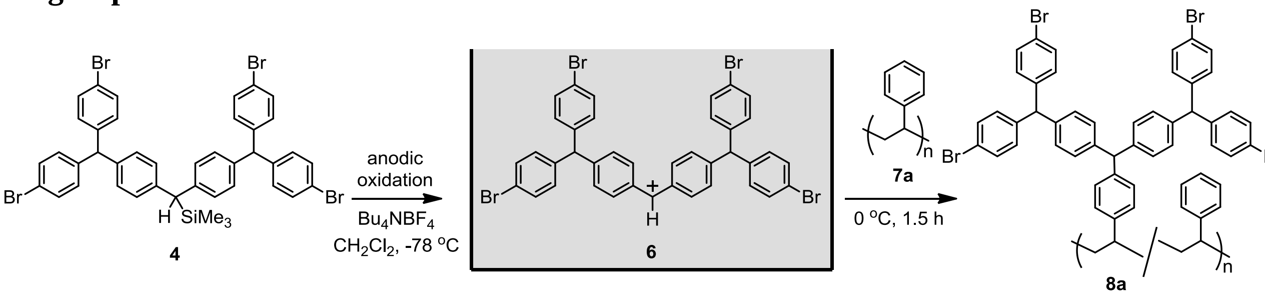

Dendronized polystyrenes **8a** and **8b** were prepared according our previously reported procedure [4]. the anodic oxidation was carried out in an H-type divided cell (4G glass filter) equipped with a carbon felt anode (Nippon Carbon GF-20-P21E, dried at 250 °C/1 mmHg for 2.5 h before use) and a platinum plate cathode (20 mm x 20 mm). In the anodic chamber were placed **4** (352 mg, 0.40 mmol) and 0.3 M Bu_4NBF_4/CH_2Cl_2 (9.0 mL). In the cathodic chamber were placed trifluoromethanesulfonic acid (100 μL, ca 1.1 mmol) and 0.3 M Bu_4NBF_4/CH_2Cl_2 (9.0 mL). The constant current electrolysis (9.0 mA) was carried out at -78 °C with magnetic stirring until 2.5 F/mol of electricity was consumed. Polystyrene (**7a**) (20 mg, Mn = 1580, Mw = 1660, PDI = 1.05) was added to the anodic chamber at -78 °C, and the mixture was stirred for 1.5 h at 0 °C. Et_3N (0.2 mL) was added at 0 °C and the resulting mixture was warmed to room temperature. After stirring at room temperature for 10 min, the solvent was removed under reduced pressure, and the residue was quickly filtered through a short column (2 x 3 cm) of silica gel to remove Bu_4NBF_4 (hexane/AcOEt 1:1 as an eluent). The solvent was removed from the combined filtrate under reduced pressure, and the thus-obtained crude product was purified with preparative GPC to obtain dendronized polymer **8a** (103 mg).

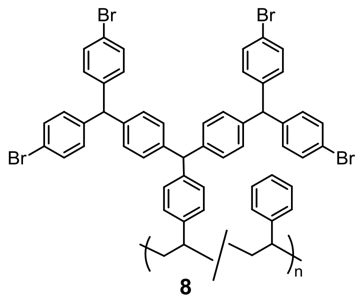

8

Dendronized polystyrene **8a**. ^{1}H-NMR (400 MHz, $CDCl_3$) δ 7.05-7.60 (m, aromatic*H*), 6.00-7.05 (m, aromatic*H*), 5.45-5.00 (m, methine*H*), 3.50-0.40 (m, alkyl*H*). ^{13}C-NMR (150 MHz, $CDCl_3$) δ 142.2, 140.6, 131.5, 130.9, 129.4, 129.1, 127.8, 120.5, 55.7, 55.2, 40.3.

Dendronized polystyrene **8b**. Generation of the cation pool of **6** from the first generation dendrimer **4** (1.78 g, 2.0 mmol) and its reaction with polystyrene (**7b**) (156 mg, Mn = 2630, PDI = 1.07) afforded dendronized polystyrene **8b** (820 mg). ^{1}H NMR (400 MHz, $CDCl_3$) δ 7.40 – 7.08 (m, aromatic*H*), 7.07 – 6.18 (m, aromatic*H*), 5.40-5.15 (m, methine*H*), 2.00 – 0.60 (m, alkyl*H*). ^{13}C-NMR (150 MHz, $CDCl_3$) δ 142.5, 142.3, 140.5, 131.5, 131.0, 129.5, 129.3, 129.0, 127.5, 120.5, 55.6, 55.2, 40.5.

6. Synthesis of the dendronized polystyrene equipped with peripheral triarylamines 9

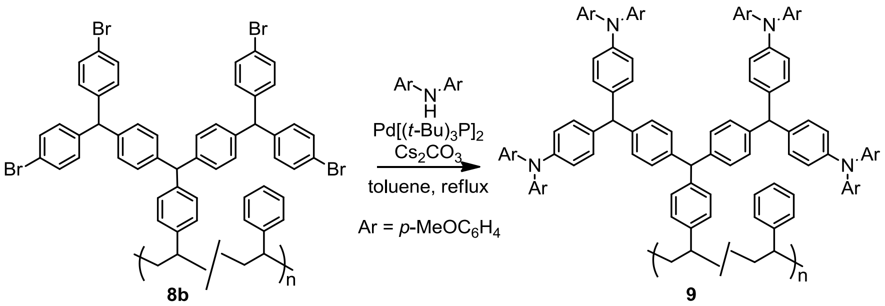

8b **9**

In a Schlenk flask, a mixture of Pd[P(^{t}Bu)$_3$]$_2$ (4 mg, 8 μmol), Cs_2CO_3 (311 mg, 0.96 mmol), bis(4-methoxy-phenyl)amine (147 mg, 0.64 mmol), and dendronized polymer **8b** (80 mg) in toluene (5 mL) was stirred under reflux condition for 69 h producing a heterogeneous mixture. The reaction mixture was treated with water and extracted with $CHCl_3$ and dried over $MgSO_4$. After evaporation of the solvent, the crude product was purified with GPC to obtain dendronized polymer **9** (112 mg). ^{1}H NMR (400 MHz, $CDCl_3$) δ 7.20 – 6.00 (m, aromatic*H*), 5.38 – 5.00 (bs, methine*H*), 3.80 – 3.40 (bs, methoxy*H*), 2.20 – 0.50 (m, alkyl*H*). ^{13}C-NMR (150 MHz, $CDCl_3$) δ 155.6, 155.4, 146.6, 142.1, 141.1, 140.8, 136.4, 129.7, 129.1, 128.5, 127.8, 126.5, 126.2, 120.5, 114.9, 114.5, 55.3, 55.1, 40.3.

7. Synthesis of the 2nd generation dendrimer without trimethylsilyl group 10

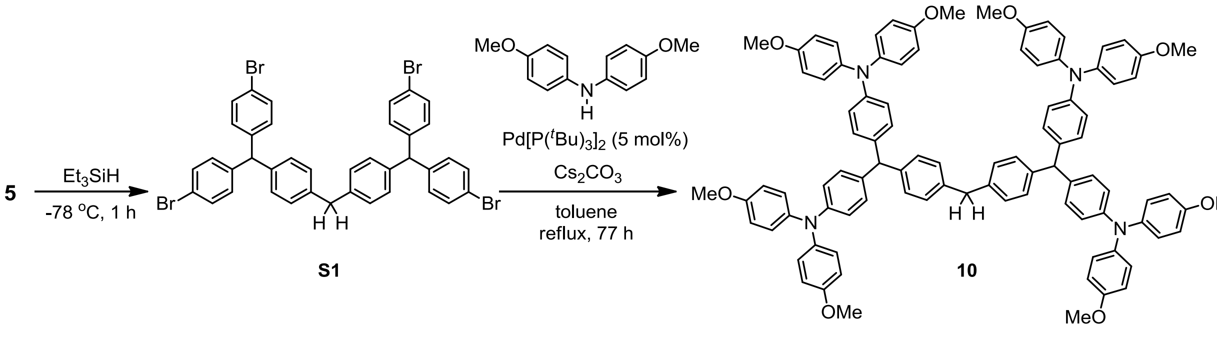

The anodic oxidation was carried out in an H-type divided cell (4G glass filter) equipped with a carbon felt anode (Nippon Carbon JF-20-P7, ca. 160 mg, dried at 300 °C/1 mmHg for 3 h before use) and a platinum plate cathode (20 mm x 10 mm). In the anodic chamber were placed a solution of **4** (895 mg, 1.0 mmol) and 0.3 M Bu_4NBF_4/CH_2Cl_2 (16 mL). In the cathodic chamber were placed trifluoromethanesulfonic acid (220 μL) and 0.3 M Bu_4NBF_4/CH_2Cl_2 (16 mL). The constant current electrolysis (40 mA) was carried out at -78 °C with magnetic stirring until 2.5 F/mol of electricity was consumed. Then triethylsilane (581 mg, 5.0 mmol) was added to the anodic chamber at -78 °C and the mixture was stirred for 1 h. The resulting mixture was treated with triethylamine (0.2 ml) at -78 °C. After stirring at room temperature for 10 min, the solvent was removed under reduced pressure and the residue was quickly filtered through a short column (2 x 3 cm) of silica gel to remove Bu_4NBF_4 by using

hexane/$CHCl_3$ = 5/1 as eluent. Removal of the solvent of the combined filtrate under reduced pressure. The crude product was purified with preparative GPC to obtain **S1** (619 mg, 76%). ^{1}H NMR (400 MHz, $CDCl_3$) δ 7.40 (d, *J*=8.4 Hz, 8 H), 7.09 (d, *J*=8.0 Hz, 4 H), 6.94-6.97 (m, 12 H), 5.40 (s, 2 H), 3.92 (s, 2 H). ^{13}C NMR (150 MHz, $CDCl_3$) δ 142.4, 140.5, 139.3, 131.5, 131.0, 129.3, 129.1, 120.5, 55.3, 41.0. HRMS (MALDI) *m/z* calcd for $C_{39}H_{28}Br_4Ag^+$: 918.7970, found: 918.7977.

In a Schlenk flask, a mixture of Pd[P(^{t}Bu)$_3$]$_2$ (0.02 mmol, 10 mg), Cs_2CO_3 (1.8 mmol, 586 mg), bis(4-methoxyphenyl)amine (1.2 mmol, 237 mg), and **S1** (0.2 mmol, 163 mg) in toluene (8 mL) was stirred reflux for 77 h producing a heterogeneous mixture. The reaction mixture was treated with water and extracted with $CHCl_3$ and dried over $MgSO_4$. After evaporation of the solvent, the crude product was purified with GPC to obtain **10** (178 mg, 63%).^{1}H NMR (400 MHz, $CDCl_3$) δ 7.00-7.08 (m, 24 H), 6.90 (d, *J*=8.4 Hz, 8 H), 6.78-6.84 (m, 24 H), 5.31 (s, 2 H), 3.91 (s, 2 H),3.77 (s, 24 H). ^{13}C NMR ($CDCl_3$, 150 MHz) δ 155.5, 146.7, 142.3, 141.2, 138.7, 136.5, 129.7, 129.3, 128.7, 126.3, 120.5, 114.5, 55.4, 55.1, 41.1. HRMS (MALDI) *m/z* calcd for $C_{95}H_{84}N_4O_8$: 1408.6284. found: 1408.6214.

8. References

(1) Saito, K.; Ueoka, K.; Matsumoto, K.; Suga, S.; Nokami, T.; Yoshida, J. *Angew. Chem., Int. Ed.* **2011**, *50*, 5153.

(2) Benkovic, S. J.; Baker, S. J.; Alley, M. R. K.; Woo, Y.-H.; Zhang, Y.-K.; Akama, T.; Mao, W.; Baboval, J.; Rajagopalan, P. T. R.; Wall, M.; Kahng, L. S.; Tavassoli, A.; Shapiro, L. *J. Med. Chem.* **2005**, *48*, 7468.

(3) Mills, N. S.; Tirla, C.; Benish, M. A.; Rakowitz, A. J.; Bebell, L. M.; Hurd, C. M. M.; Bria, A. L. M. *J. Org. Chem.* **2005**, *70*, 10709.

(4) Nokami, T.; Watanabe, T.; Musya, N.; Suehiro, T.; Morofuji, T.; Yoshida, J. *Tetrahedron* **2011**, *67*, 4664.

9. ^{1}H NMR and ^{13}C NMR spectra

^{1}H NMR (400 MHz)

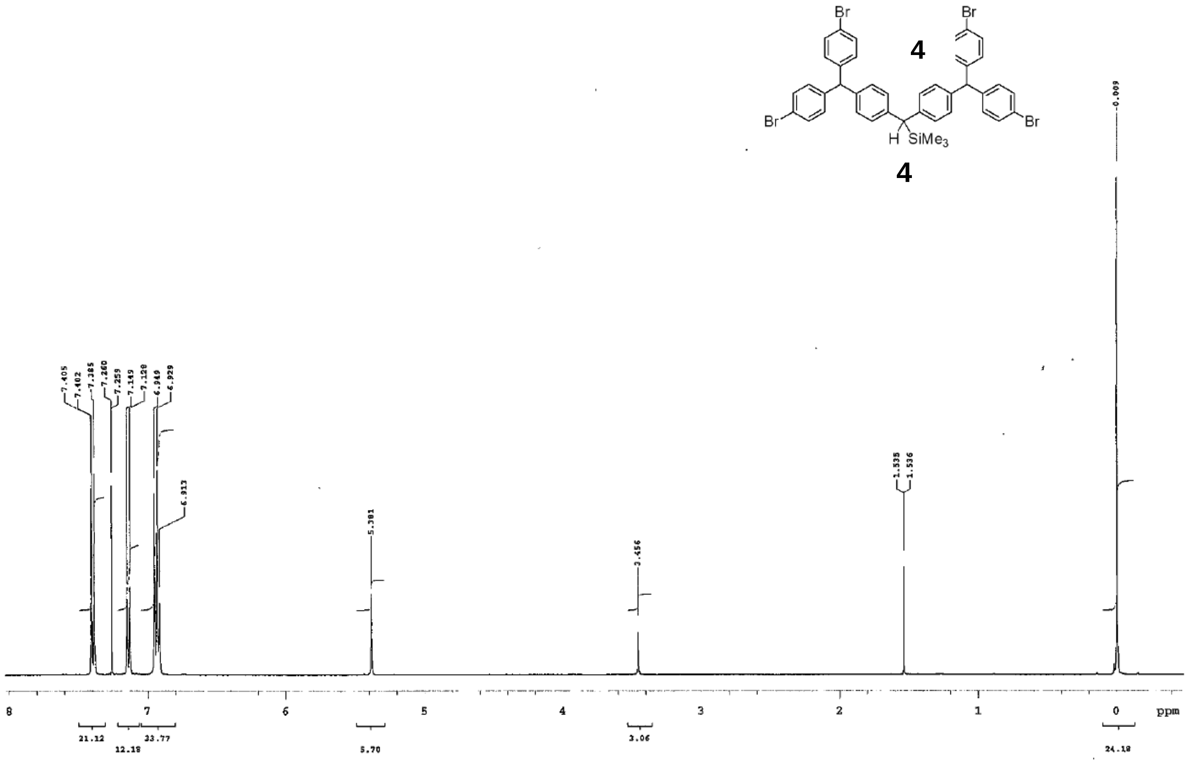

^{13}C NMR (100 MHz)

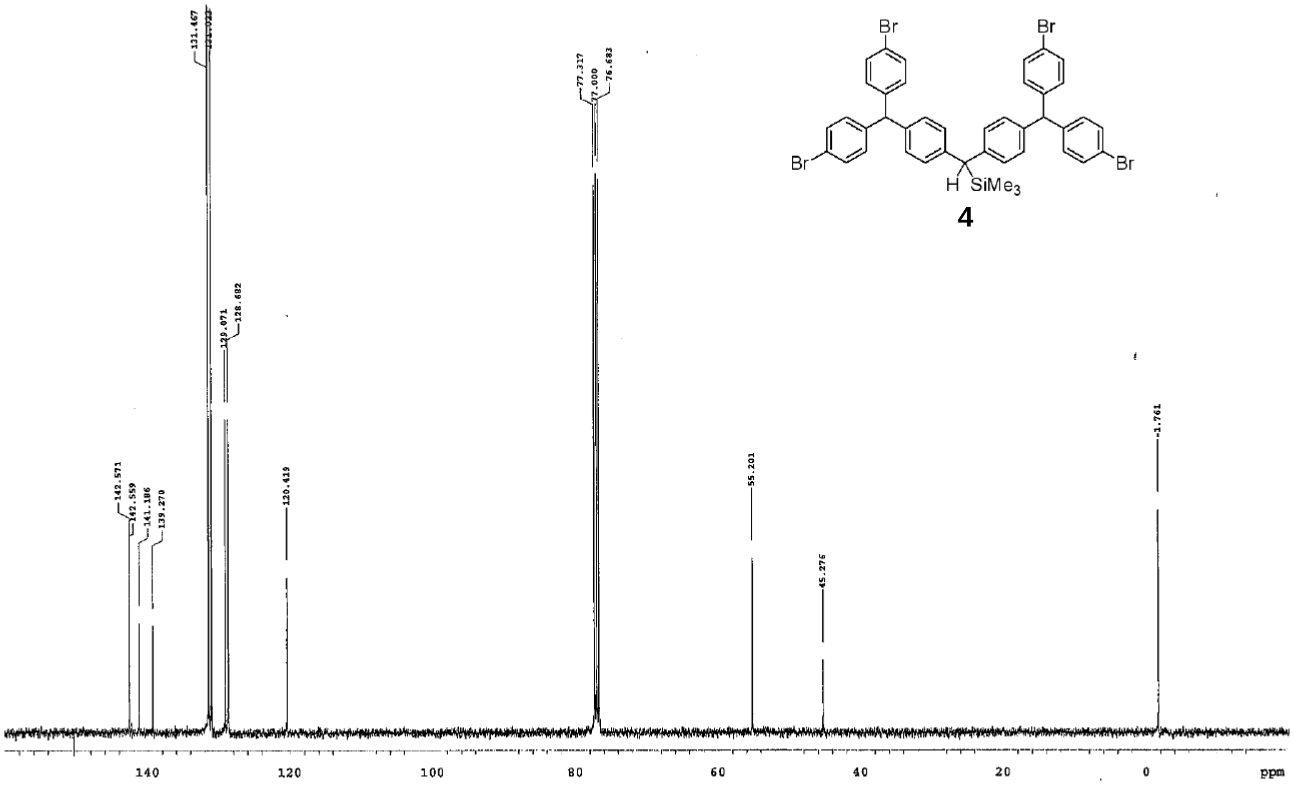

^{1}H NMR (600 MHz)

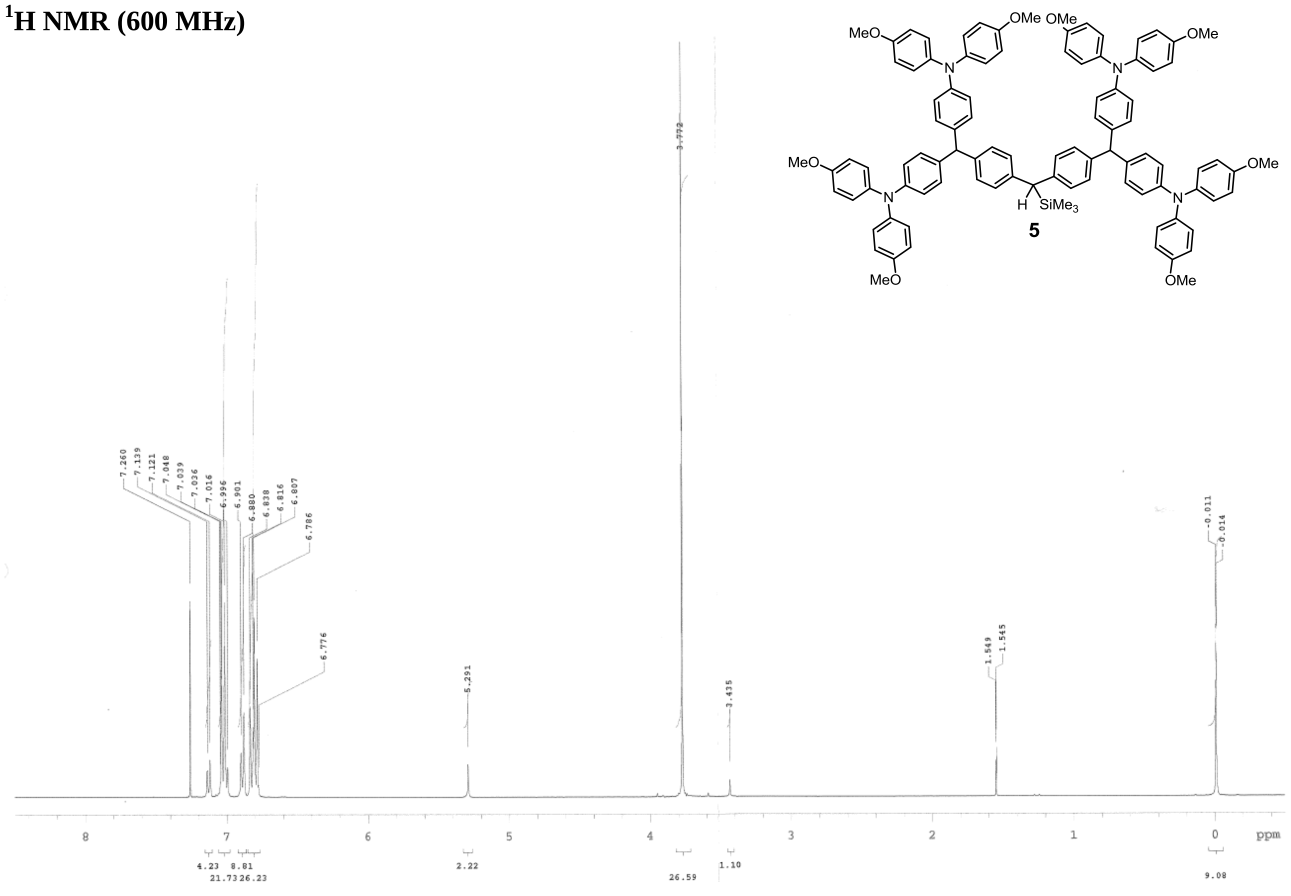

^{13}C NMR (150 MHz)

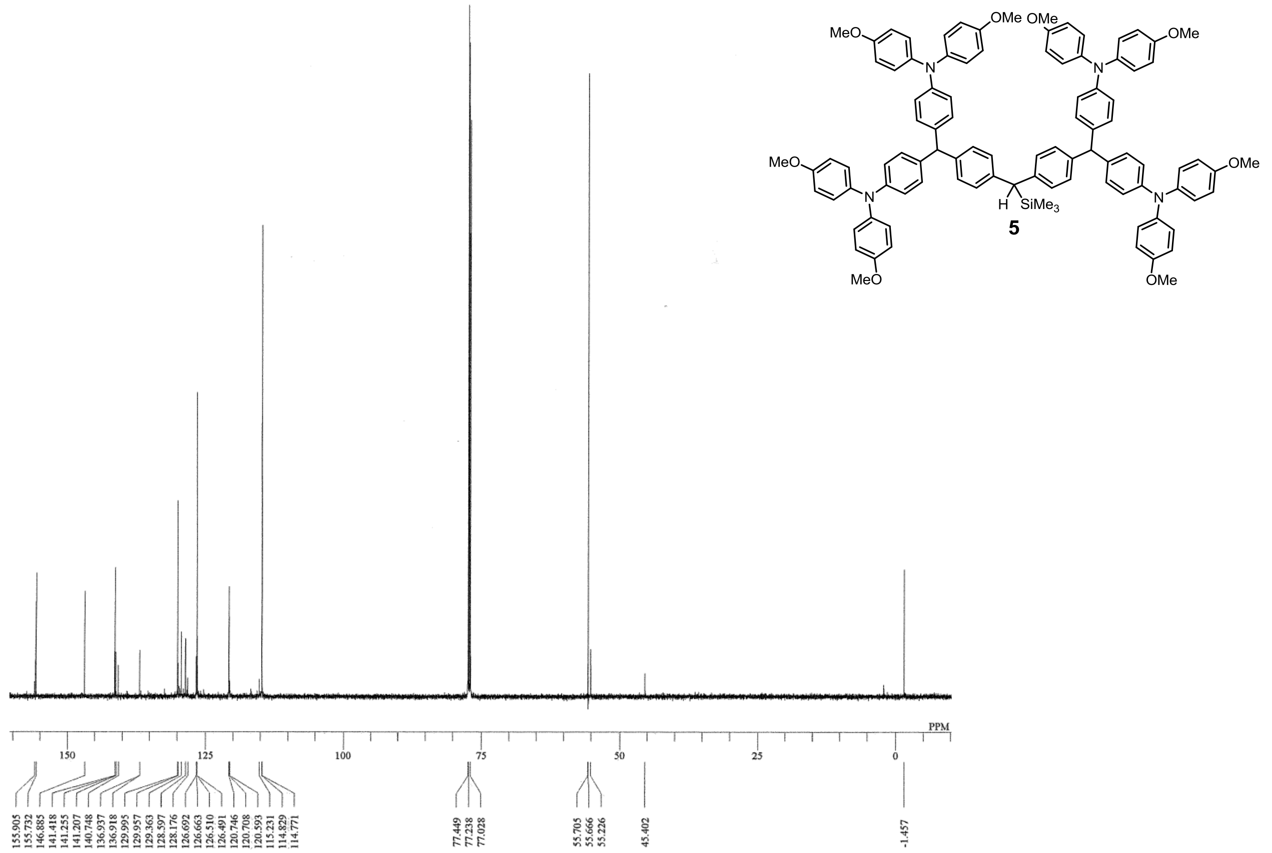

^{1}H NMR (600 MHz)

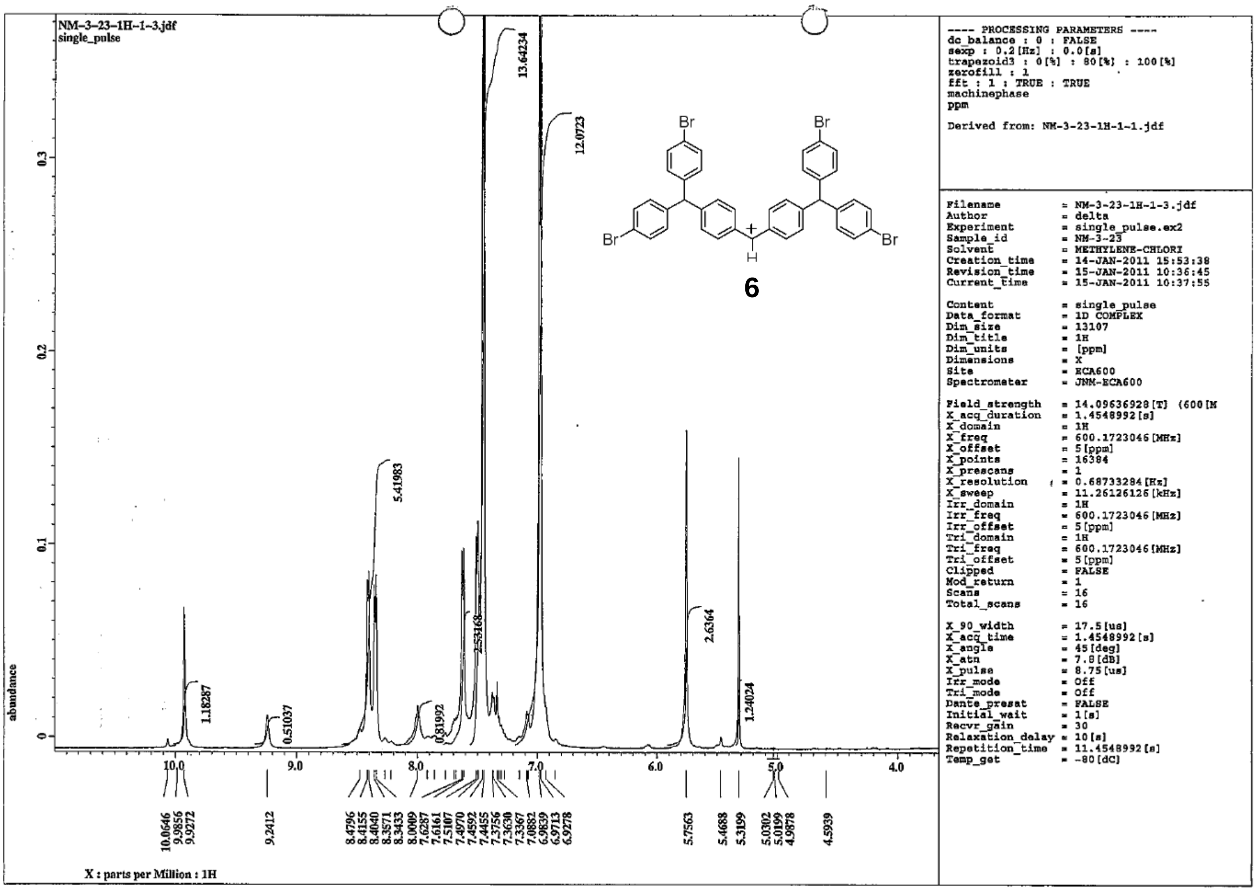

^{13}C NMR (150 MHz)

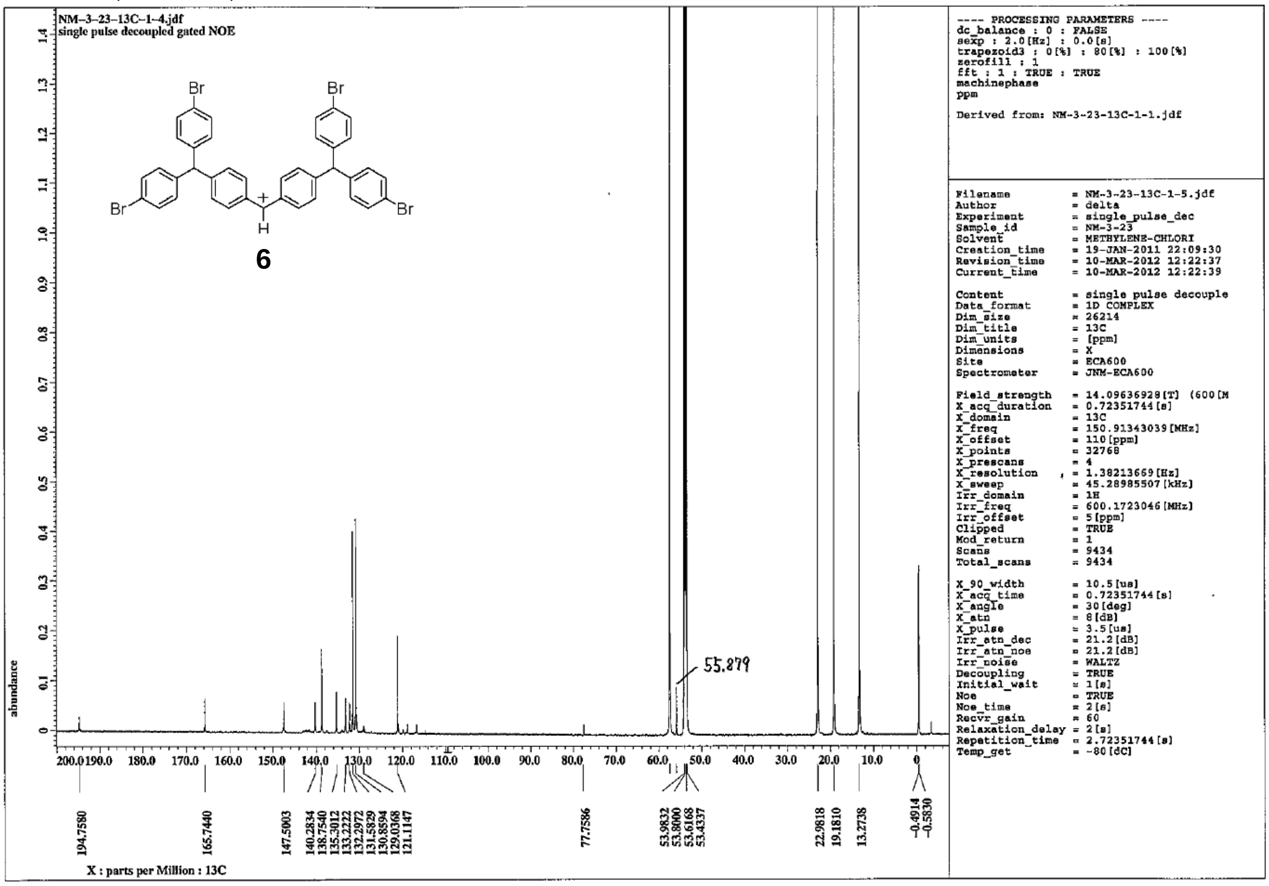

HMQC

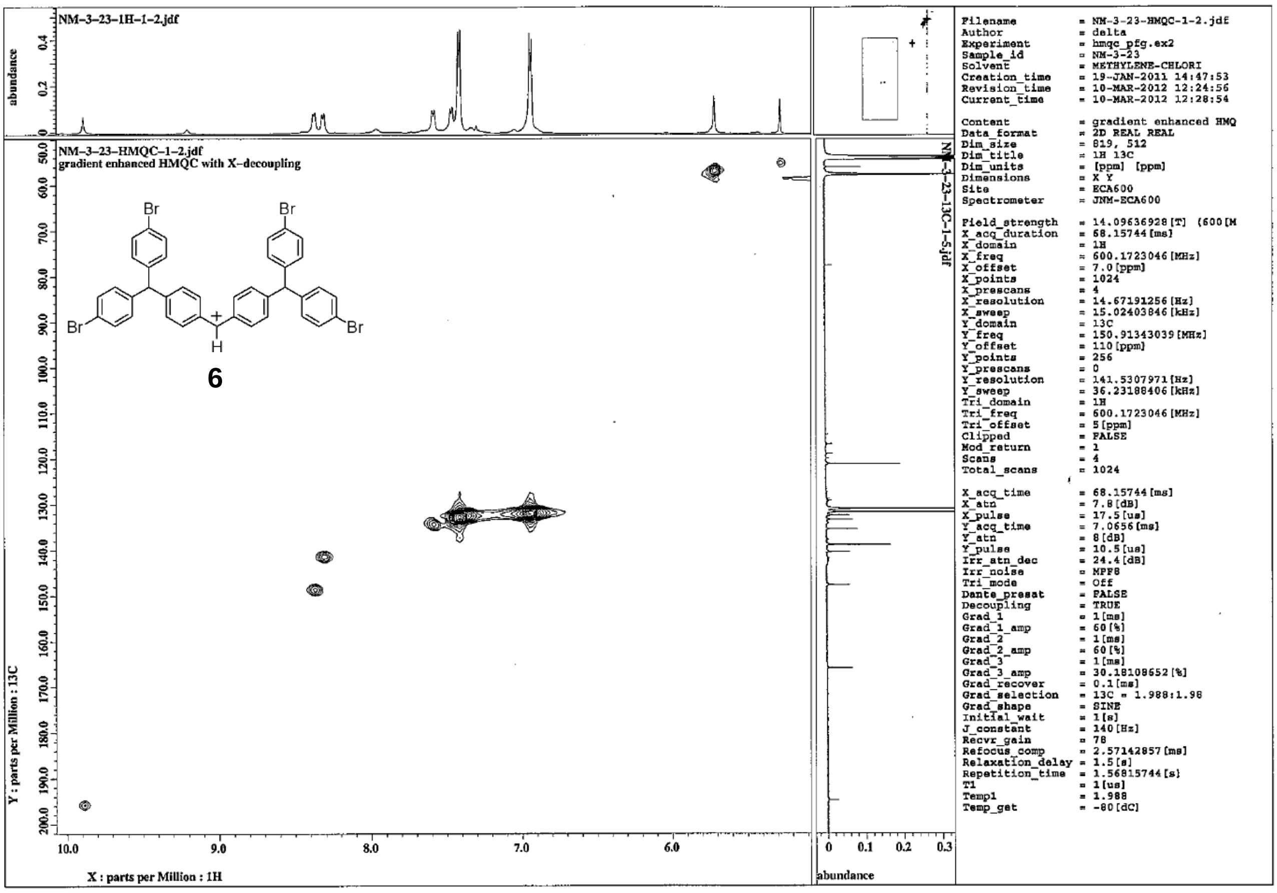

^{1}H NMR (600 MHz)

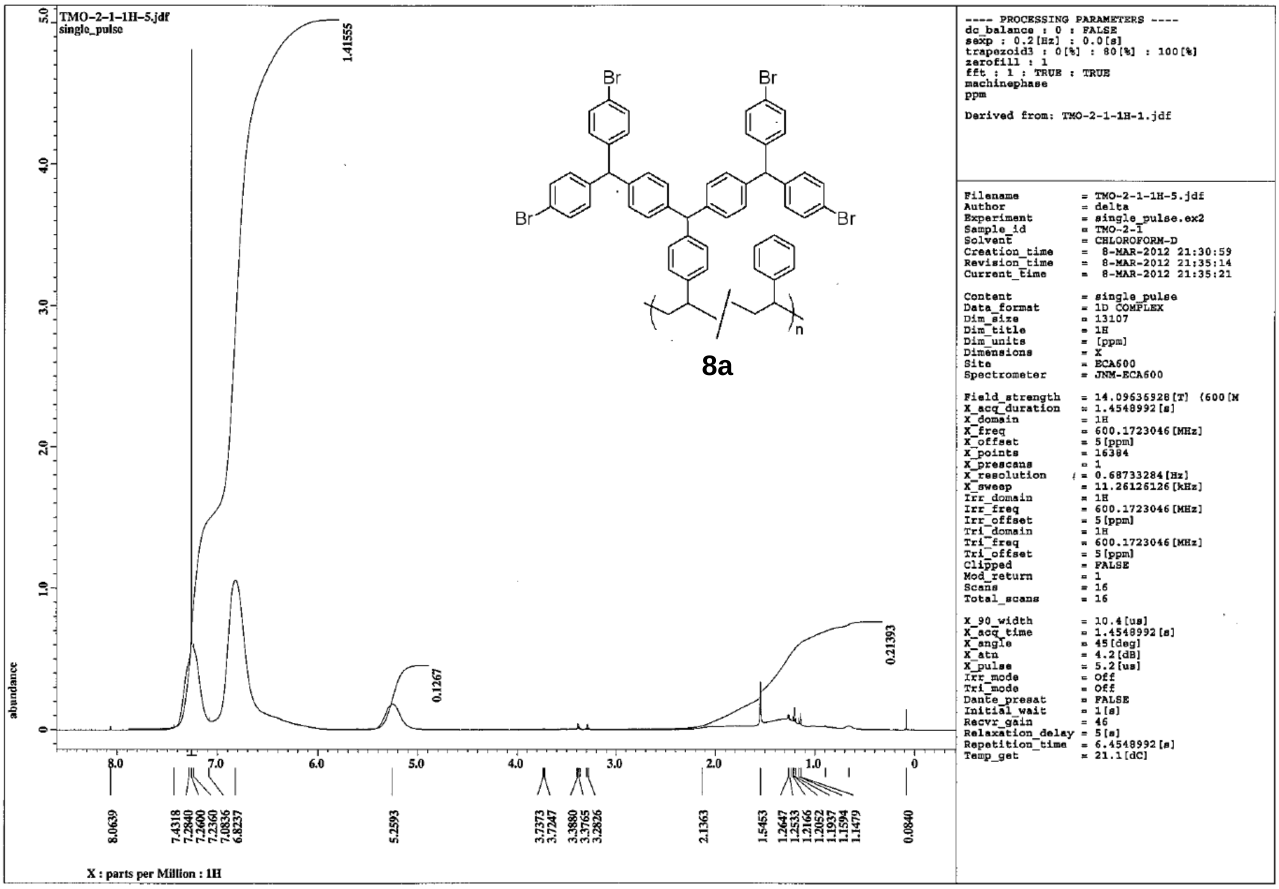

^{13}C NMR (150 MHz)

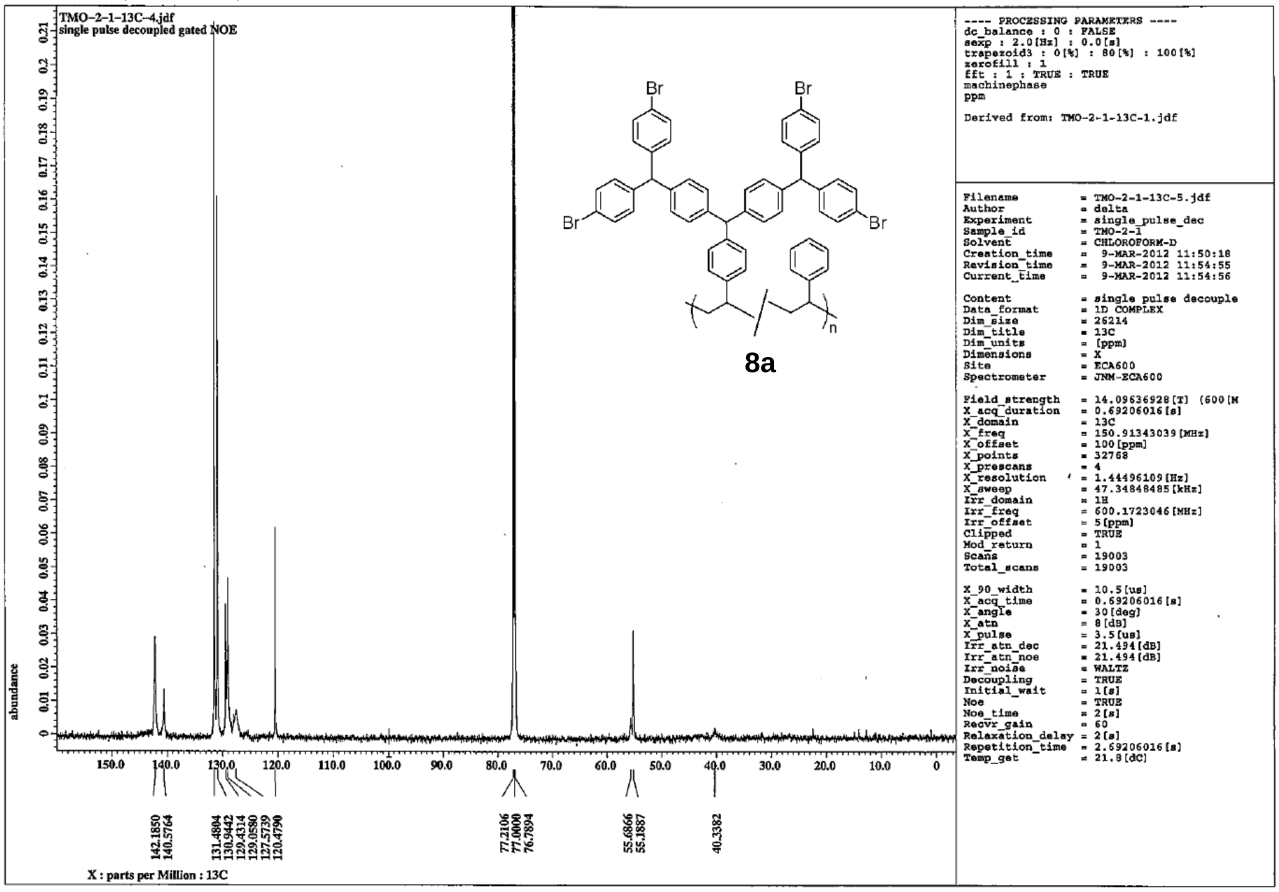

^{1}H NMR (400 MHz)

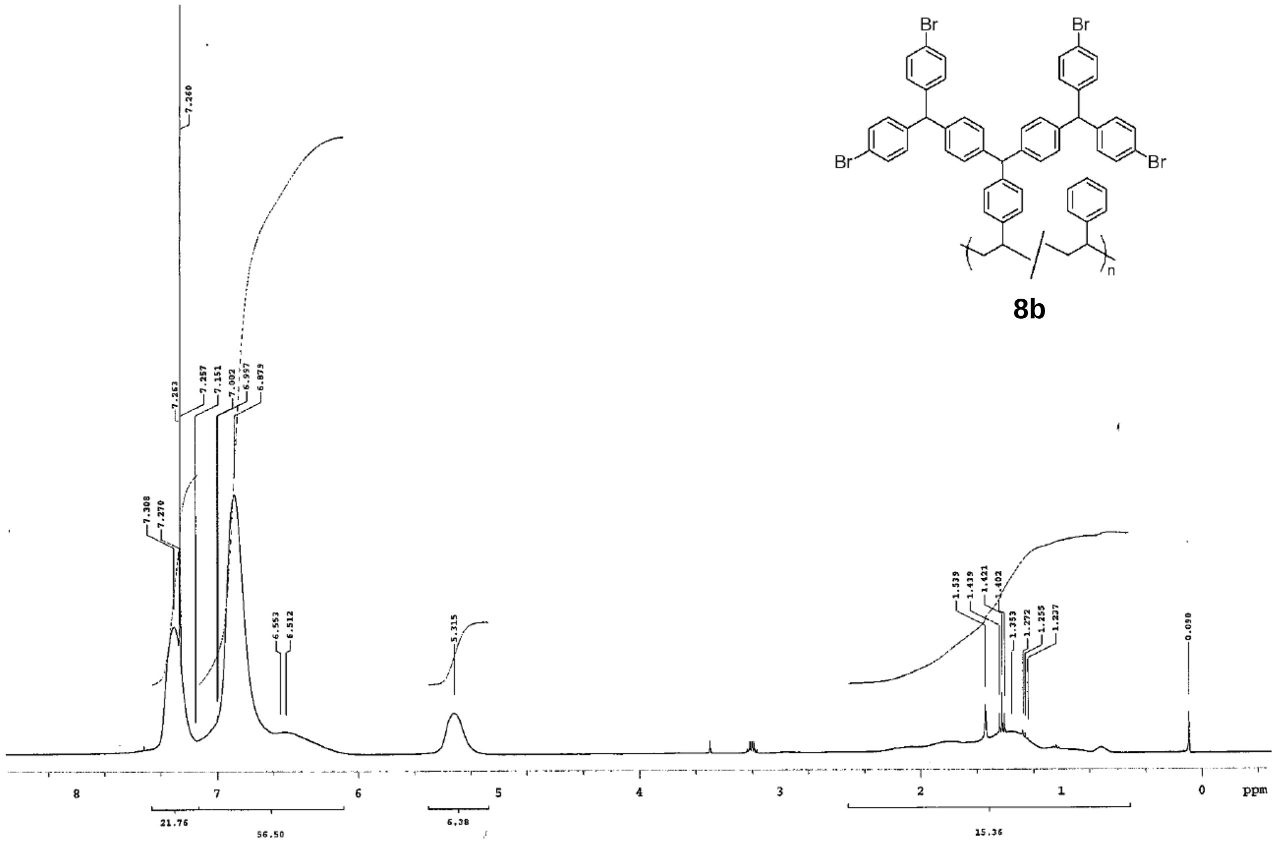

^{13}C NMR (150 MHz)

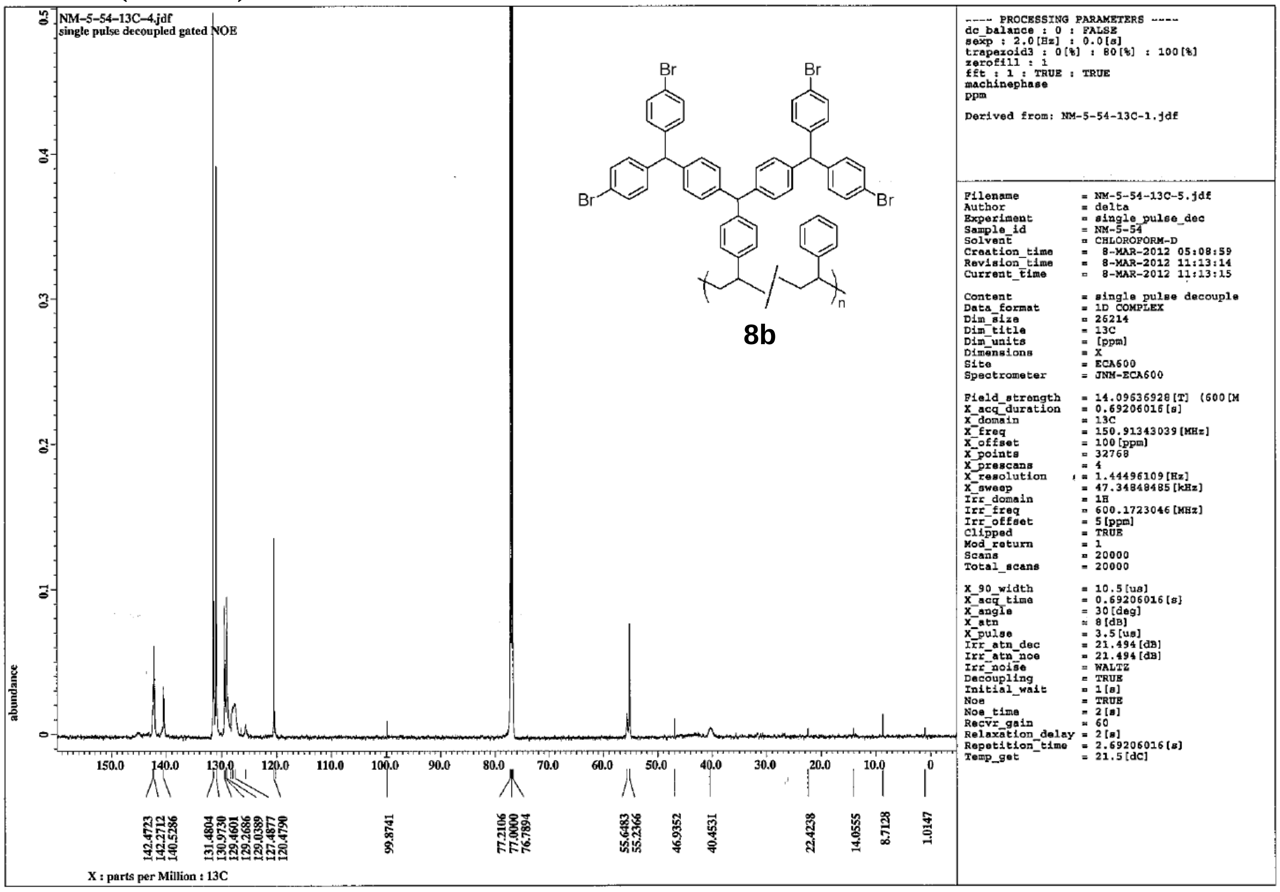

^{1}H NMR (400 MHz)

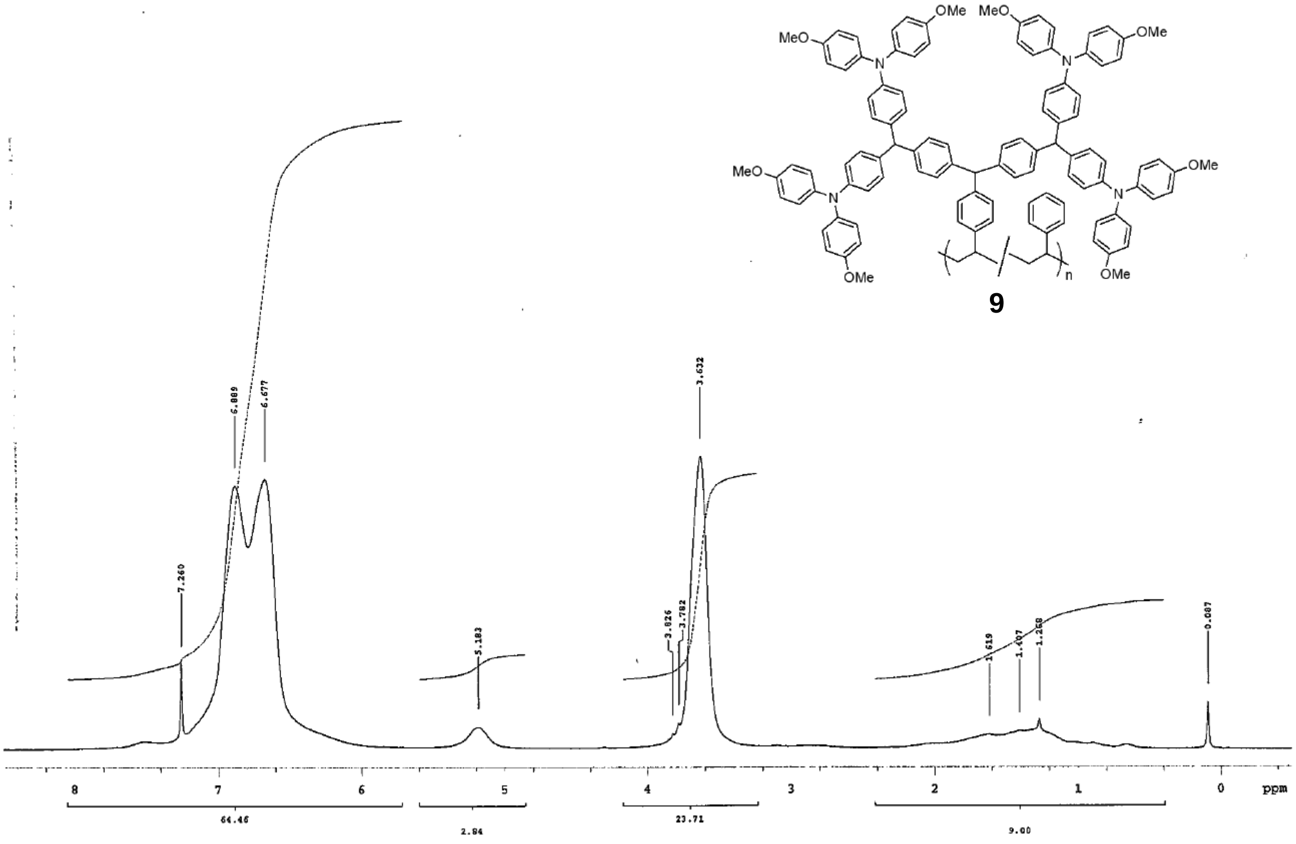

^{13}C NMR (150 MHz)

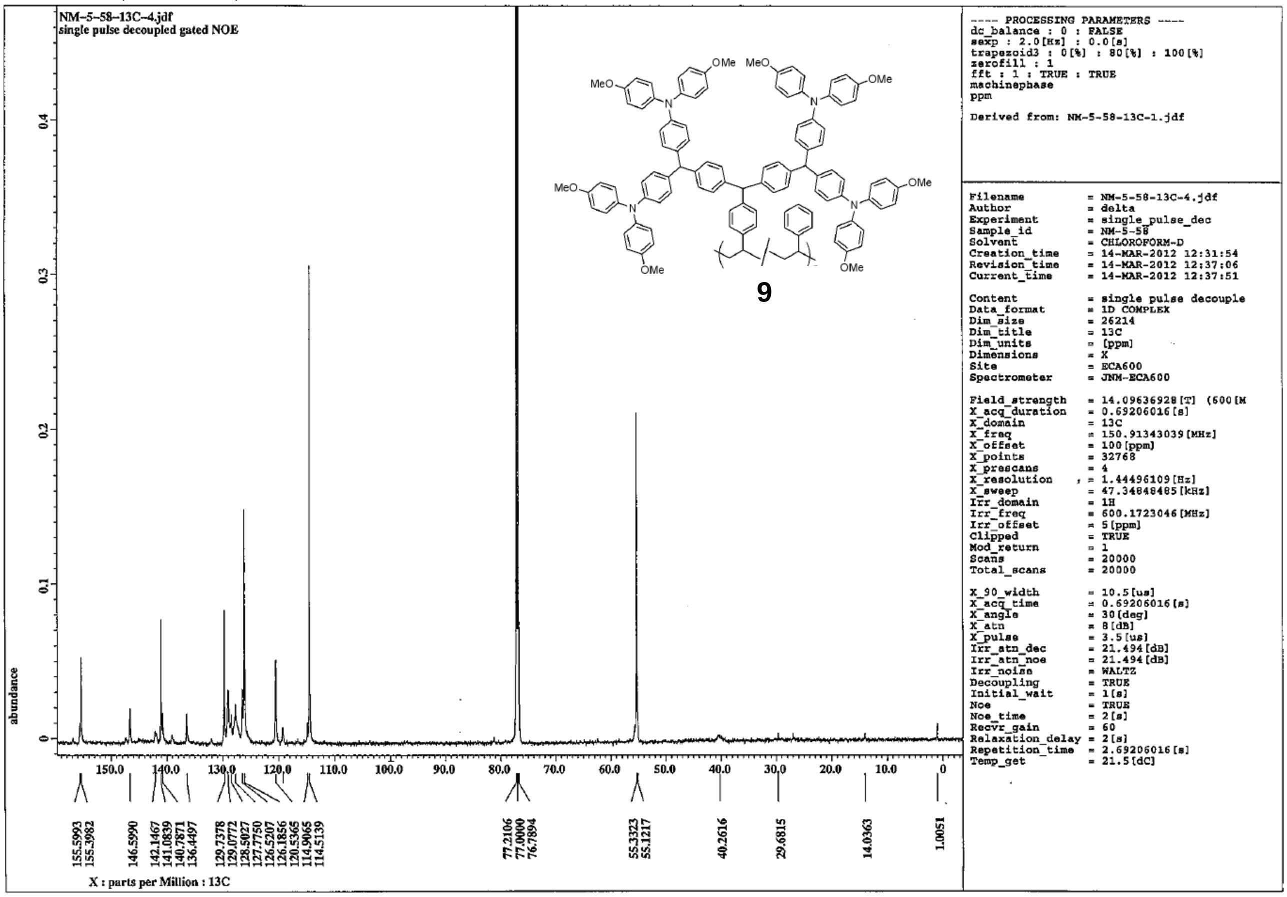

^{1}H NMR (400 MHz)

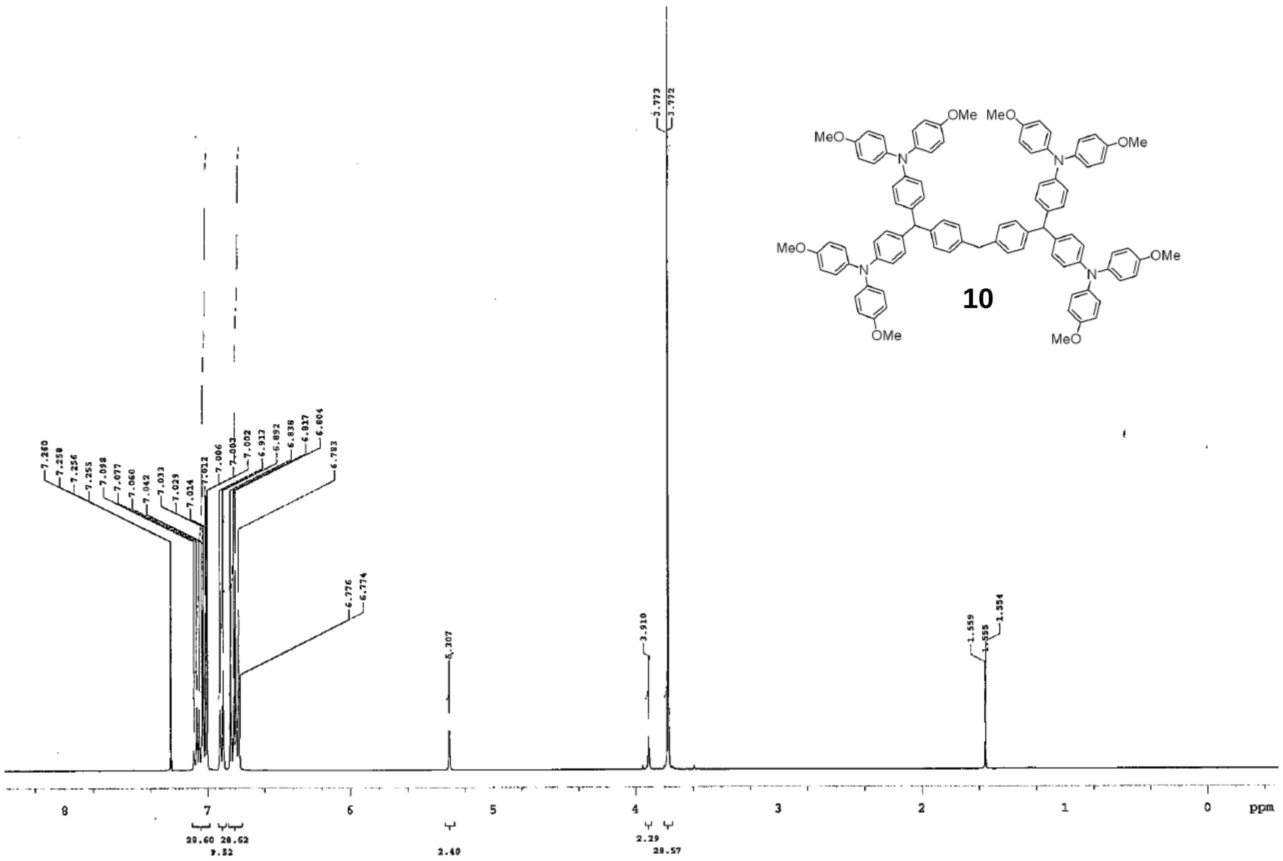

^{13}C NMR (150 MHz)

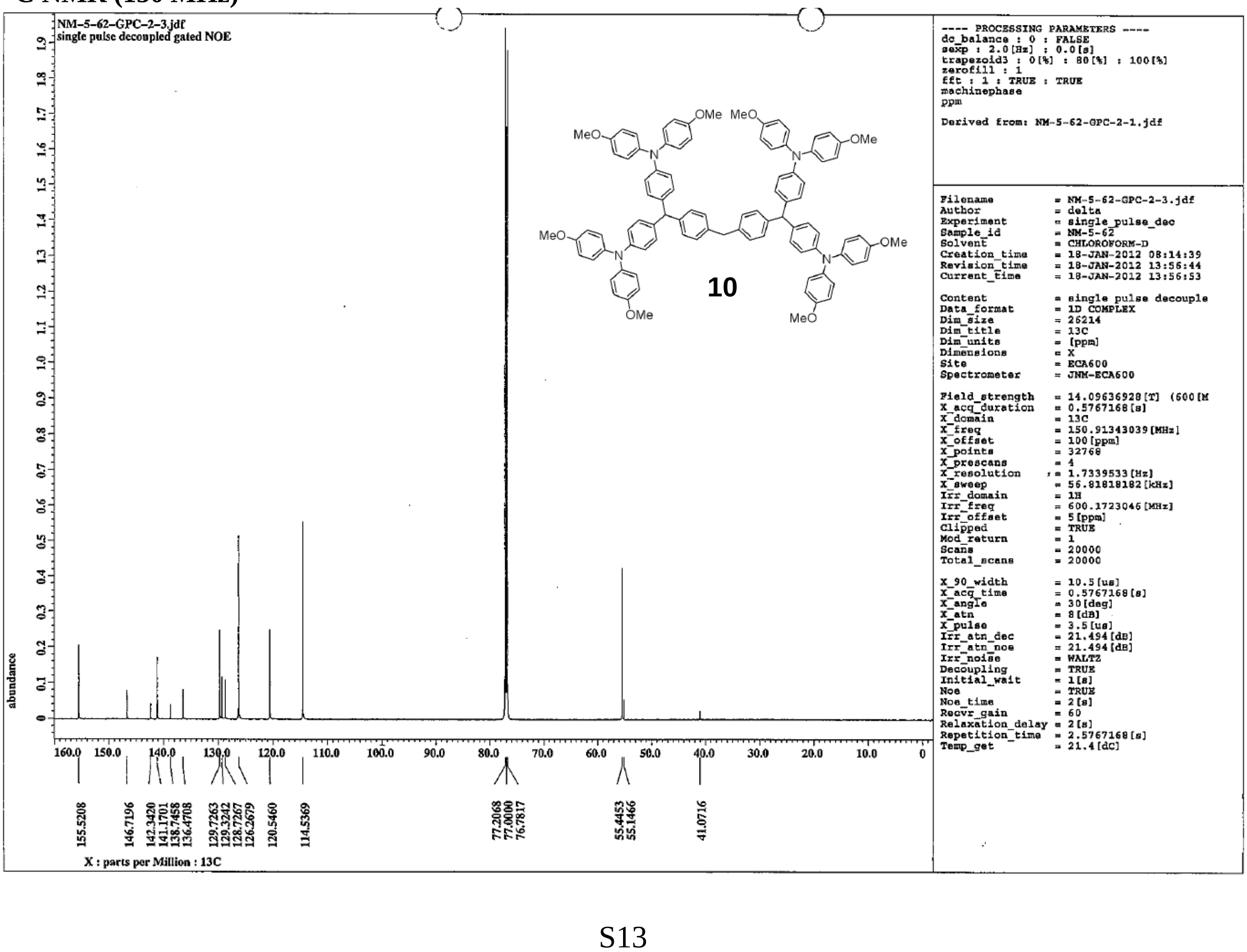

^{1}H NMR (400 MHz)

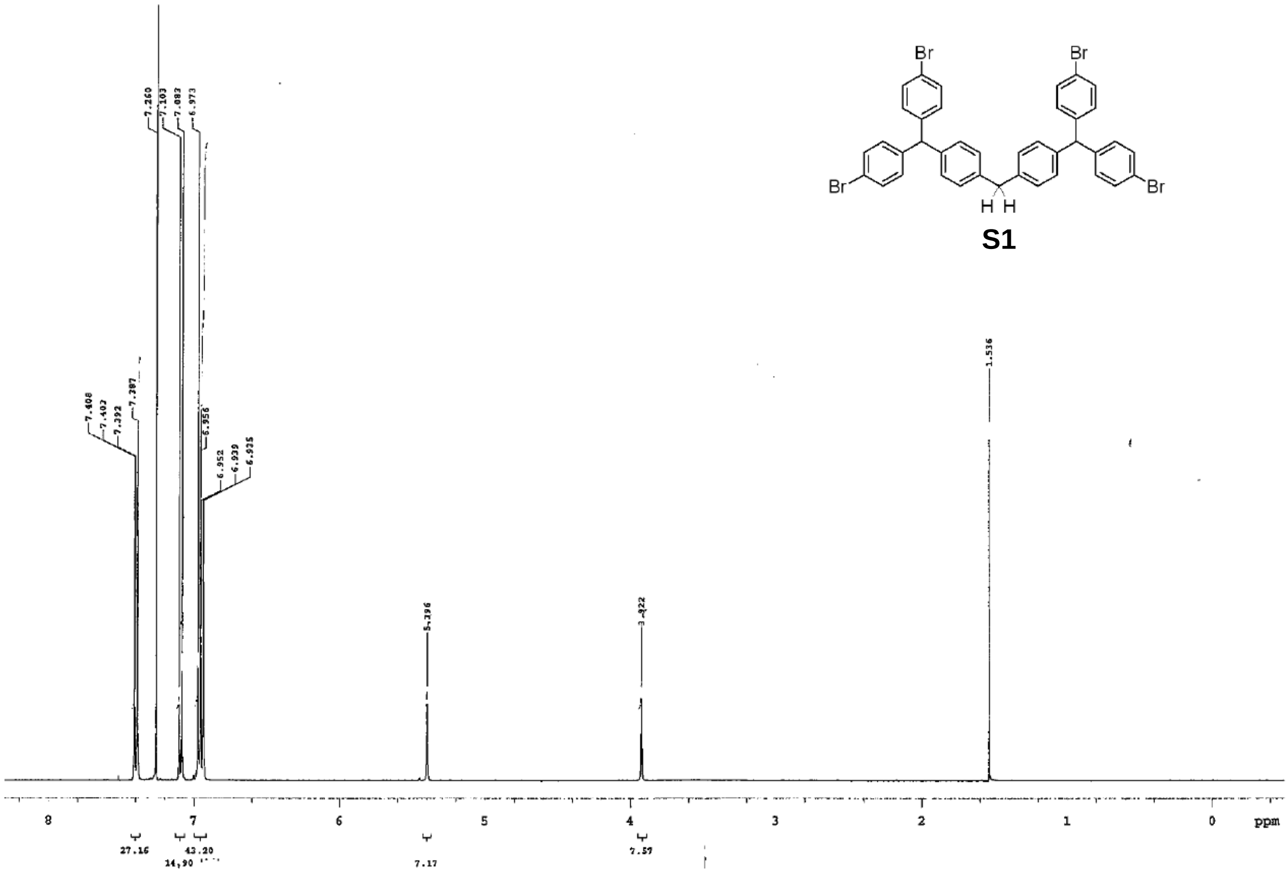

^{13}C NMR (150 MHz)

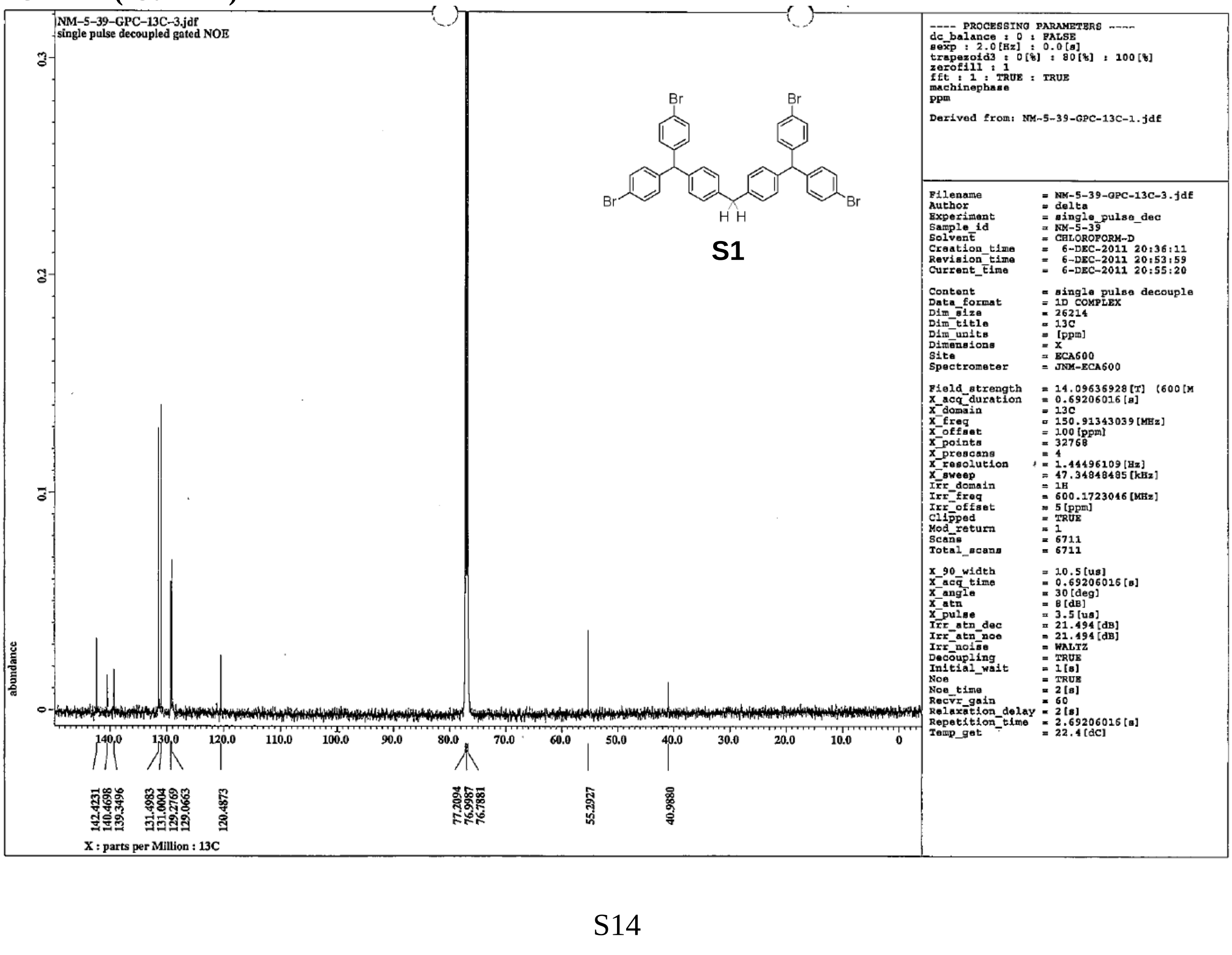

Supplement: File 1 — Experimental procedures for the synthesis of new compounds and spectral data of new compounds including 1H NMR, 13C NMR, and HMQC spectra. [file Beilstein_J_Org_Chem-10-3097-s001.pdf]
